# Human-Centric Lighting: Foundational Considerations and a Five-Step Design Process

**DOI:** 10.3389/fneur.2021.630553

**Published:** 2021-01-27

**Authors:** Kevin W. Houser, Tony Esposito

**Affiliations:** ^1^School of Civil and Construction Engineering, Oregon State University, Corvallis, OR, United States; ^2^Advanced Lighting Team, Pacific Northwest National Laboratory, Portland, OR, United States; ^3^Lighting Research Solutions LLC, Cambridge, MA, United States

**Keywords:** human-centric lighting, lighting quality, non-visual action of light, circadian light effects, alertness

## Abstract

At its best, human-centric lighting considers the visual and non-visual effects of light in support of positive human outcomes. At its worst, it is a marketing phrase used to healthwash lighting products or lighting design solutions. There is no doubt that environmental lighting contributes to human health, but how might one practice human-centric lighting given both the credible potential and the implausible hype? Marketing literature is filled with promises. Technical lighting societies have summarized the science but have not yet offered design guidance. Meanwhile, designers are in the middle, attempting to distinguish credible knowledge from that which is dubious to make design decisions that affect people directly. This article is intended to: (1) empower the reader with fundamental understandings of ways in which light affects health; (2) provide a process for human-centric lighting design that can dovetail with the decision-making process that is already a part of a designer's workflow.

## Introduction

Human-centric lighting is an idiom intended to describe lighting solutions that considers the traditional elements of lighting quality that are rooted in human vision while simultaneously incorporating new insights about the non-visual effects of light. Recently, Houser et al. (1) outlined the rise of human-centric lighting and its current status in lighting. That manuscript describes a range of visual and non-visual responses and the eye-brain pathways that drive them, outlines the agreed upon science that can inform the practice of human-centric lighting, and offers general guidance for the practice of human-centric lighting. That work asserts that human-centric lighting, also called integrative lighting (2, 3), is not a product feature, and that lighting products that claim to improve sleep or performance should be met with skepticism.

Instead, human-centric lighting begins with effective prioritization of design goals and is an outcome of good decision making at every step in the lighting design process. Human-centric lighting begins with project conceptualization, and continues through prioritization of design goals, architectural design (including daylighting design), lighting equipment specification, commissioning, and operation of the lighting systems. Successful implementation requires buy-in from all stakeholders involved in building design, construction, and operation, including occupants.

The stakeholders with the most at stake are occupants, since it is their health, well-being, and cognitive performance that we are concerned about. Yet, designers cannot influence light exposure when occupants are elsewhere, such as at home, in transit, or in other buildings. Individual outcomes will vary based on individual lifestyles and habits. As will be expanded upon later, the best that a designer can do is to provide the proper light at the proper time for the projects that they design.

Since many American adults spend about 90% of their time in buildings (4, 5), we believe that human-centric lighting should almost always be a part of a designer's scope and a building owner's desires. For the many people that spend their time indoors, days are dimmer and nights are brighter than would be experienced in nature (6–8). Thus, while natural light from the sky and sun is ideal for many health outcomes associated with light, electric lighting has a critical role in supporting human health in the modern world.

Implementing a successful human-centric lighting solution is complex. The goal of this work, therefore, is to outline the early stages of the design process for projects where human-centric lighting is deemed important. We offer a five-step process that will help organize information gathering and decision-making. Though no standard for biologically effective lighting has gone through the full consensus process required by ANSI, ISO, or IEC, we consider compliance with WELL v2 (9) and UL Design Guideline 24480 (10) as they may be appropriate for some projects. In support of informed decision making, we summarize relevant responses of people to light and lighting. Our review is intended to not overwhelm the reader with every study that links light with human physiology. Instead, we offer enough background to describe the unequivocal physiological responses to light, including why such responses may not always affect real-world outcomes. The content covered in this manuscript is complemented by reviews by others (1, 11–16).

## Foundational Considerations

The goal of this section is to succinctly introduce the principles of human-centric lighting in support of conversational understanding of the most relevant considerations for lighting design.

### The Stimulus Response Relationship Between Light and Human Outcomes

**Figure 1:**
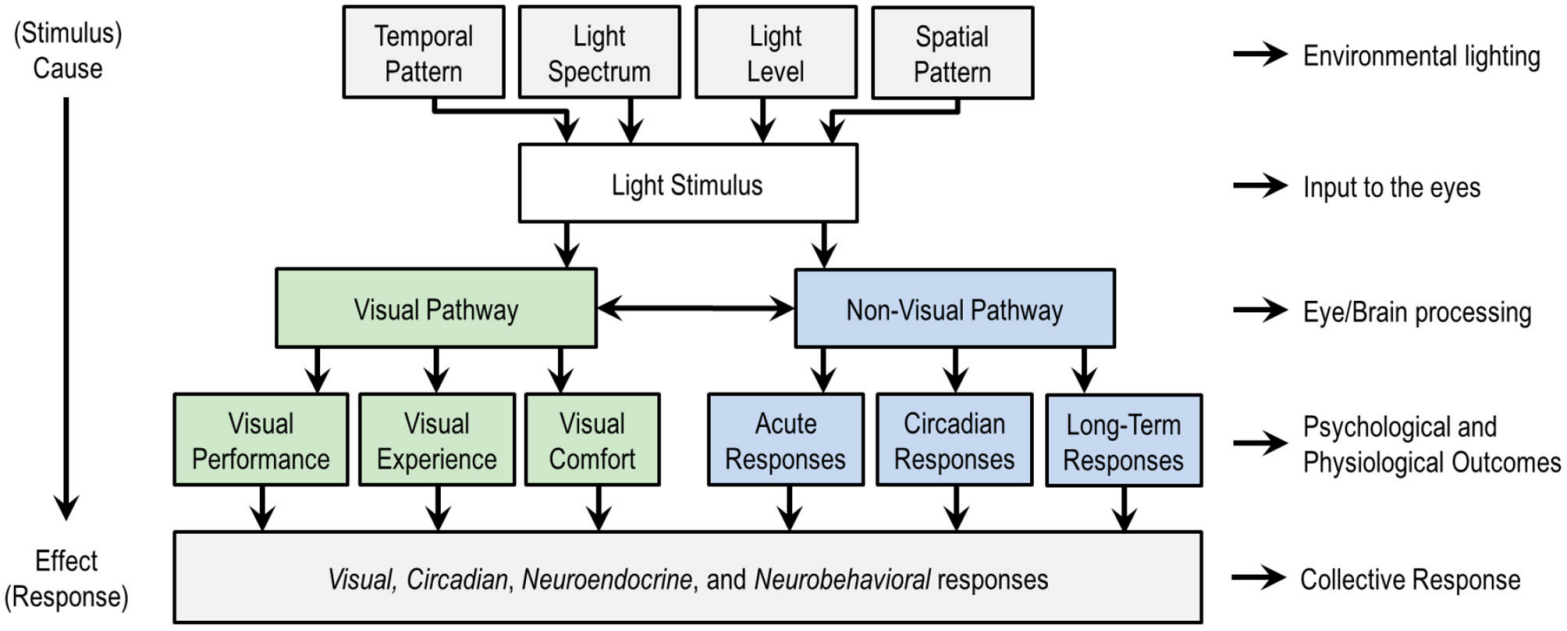
An overview of the stimulus (top) response (bottom) relationship between light and human responses, with a schematic subdivision of the visual and non-visual responses. At the top level, the temporal pattern relates the timing and duration of exposure to a light stimulus, spatial pattern refers to the spatial distribution of light in the three-dimensional light field, light spectrum refers to the spectral power distribution (SPD) that governs color qualities, and light level refers to the quantity of light in radiometric or photometric units. These four factors contribute to the biological potency of the light stimulus. Designers often vary factors together, though any of the factors can be disengaged from any other. Researchers usually vary just a small number of factors, sometimes only one, to isolate cause and effect. Non-lighting factors not shown such as age and chronotype moderate the effects of light on people and are important in practice. This figure only considers the effects of light through the eyes; the effects of optical radiation on or through the skin are not considered here. Figure inspired by de Kort and Veitch (17).

Figure 1 is a schematic of the stimulus response relationship between light and human outcomes. The top four categories are lighting variables that can be manipulated in the design of a light stimulus, whether in lighting experiments or in the design of the built environment. These categories are temporal pattern, light level, light spectrum, and spatial pattern.

These four aspects of the light stimulus can be manipulated to influence two categories of human responses to light, visual and non-visual, as illustrated in the middle of Figure 1. A *visual response* is an eye-brain response that enables sight, contributing to visual performance, the visual experience including emotional responses, and visual comfort (or discomfort, as with glare). Non-visual responses might also be called non-image forming (NIF) effects of light, biological responses, or physiological responses. CIE adopted the phrase ipRGC-influenced light responses (18) and IES uses the phrase visual, circadian, neuroendocrine, and neurobehavioral responses (19). *Circadian responses* are internal biological processes that occur on a roughly 24-h period, such as the sleep-wake cycle. *Neuroendocrine responses* refer to how the brain regulates hormones, such as expression of melatonin. *Neurobehavioral responses* refer to the relationship between the action of the nervous system and human behavior. The IES terminology is adopted in the bottom of Figure 1 because this collection of responses encompasses all the ways that light may affect people through the eyes. Table 1 provides examples of physiological and psychophysical responses that are important to human health, well-being, cognition, and performance, and which may be influenced by light and lighting over different time periods.

**Table 1:** Examples of psychophysical and physiological responses that are influenced by light and lighting.

**Time Course**	**Psychophysical**	**Physiological**
Immediate (seconds or minutes)	• Brightness perception • Visual amenity • Visual discomfort • Attention response	• Pupil size • Acute melatonin suppression • Luminance adaptation • Short-term chromatic adaptation
Delayed (hours, days, or weeks)	• Mood • Cognition • Motivation	• Circadian phase shift • Sleep quality • Long-term chromatic adaptation
Long-Term (months or years)	• Productivity • Depression	• Stress • Poor health • Seasonal affective disorder • Depression

*Psychophysical responses are largely though not exclusively driven by the visual pathway and physiological responses are largely though not exclusively driven by the non-visual pathway (see also Figure 1). Every item in this table is subject to influence by non-lighting factors. For example, even an immediate effect like pupil size may be affected by signals from other senses. The longer the delay between the stimulus and the response, the more opportunity there is for other factors to influence the response*.

The four categories of lighting variables identified in the top row of Figure 1 have unequal influences on non-visual responses to light, and thus contribute unequally to the biological potency of a light stimulus. *Biological potency* refers to the strength of influence of a light stimulus on a human biological response.

The temporal pattern is most important to non-visual responses because the brain tells time by observing nature's daily pattern of light and dark (20). The same light stimulus may be beneficial at one time of day but detrimental at another. For day-active people—that is, people who are active during the day, relatively less active in the evening, and sleep at night—bright light in the morning and during the day will support health, whereas bright light in the evening may delay the onset of sleep and be detrimental to health.

At any given time, the biological potency of a light stimulus can be altered first by adjusting light level and second by adjusting light spectrum. Brighter light with proportionally more short-wavelength radiation, coinciding with the melanopsin action spectrum, is more biologically potent than dimmer light with proportionally less short-wavelength radiation that coincides with the melanopsin action spectrum. At constant illuminance, broadband sources with a higher CCT are often more biologically potent than those with a lower CCT, though this depends upon how biological potency is quantified. CCT, however, is a one-dimensional reduction of a light source SPD that cannot reliably predict biological potency, a limitation that is especially apparent for color-mixed LEDs (21, 22) or any other SPD with pronounced peaks and valleys.

With respect to the spatial pattern of light, light exposure on the lower retina more effectively suppresses melatonin than light on the upper retina (23), and light exposure on the nasal side of the retina is more biologically potent than light exposure on the temporal side of the retina (24, 25). In building interiors, these effects are likely dwarfed by gaze direction. Looking toward a bright window will produce a more biologically potent light stimulus than looking toward a dimly lit interior wall. Lighting criteria for vision are at task locations and are often oriented horizontally (26), whereas criteria for circadian lighting design are at the plane of the occupant's eyes and are oriented vertically (9, 10, 27). There are opportunities for novel lighting solutions that balance the need to deliver light at the plane of an observer's eyes without causing visual discomfort.

### Quantifying the Biological Potency of Light for Human-Centric Lighting Design

Visual and non-visual responses are driven by photoreceptors that reside in the retina and send signals to the brain. Visual responses in humans are largely driven by the rods and cones. Non-visual responses are largely driven by intrinsically photosensitive retinal ganglion cells (ipRGCs) (28, 29). The ipRGCs combine their own intrinsic response to light with extrinsic inputs from the rods and cones (30). Though the understanding of the balance between extrinsic and intrinsic signaling in the various ipRGC subtypes (31) is incomplete, it is known that all photoreceptors contribute to both visual and non-visual responses.

There are two prevalent methods for quantifying light as a non-visual stimulus: (1) based on the spectral response of the photopigments in the rods, cones, and ipRGCs (18, 32), (2) based on nocturnal suppression of the hormone melatonin (33–36).

Equivalent Melanopic Lux (EML) introduced by Lucas et al. (32) is computed as the product of Photopic Illuminance, E, and the Melanopic Ratio, R, where:

(1) $$\begin{array}{r} \text{EML}=\left(\text{E}\right)\left(\text{R}\right) \end{array}$$

EML is expressed in units of melanopic lux (m-lux), which is not recognized by the International System of Units (SI). R is computed as the ratio of a light source's melanopsin-activating radiation to its photopic-activating radiation, multiplied by 1.218, which guarantees that R = 1.0 for the Equal Energy illuminant. R is unitless and ranges from ~0.45 to 1.70. On average, light sources with a higher CCT also have a higher value for R.

CIE has proscribed the use of EML and has proposed an SI compliant quantity as its replacement (18). Melanopic Equivalent Daylight Illuminance, melanopic EDI, $E_{\text{v},\text{mel}}^{\text{D}65}$, or “mel-EDI” for short, is the illuminance of standard daylight (D65), at a point, that provides equal melanopic irradiance as the test source. For example, a mel-EDI of 100 lx means that the light source under evaluation produces the same amount of melanopsin-activating radiation as 100 lx of daylight at 6,500 K. It is computed as the product of photopic illuminance, *E*_v_, and the melanopic Daylight Efficacy Ratio, melanopic DER, $\gamma_{\text{v},\text{mel}}^{\text{D}65}$, or “mel-DER” for short, where:

(2) $$\begin{array}{r} E_{\text{v},\text{mel}}^{\text{D65}}=(E_{\text{v}})\left(\gamma_{\text{mel},\text{v}}^{\text{D65}}\right) \end{array}$$

$E_{\text{v},\text{mel}}^{\text{D}65}$ (mel-EDI) is expressed in units of lux, which is an SI-compliant unit. $\gamma_{\text{v},\text{mel}}^{\text{D}65}$ (mel-DER) is computed as the ratio of a test source's melanopic efficacy of luminous radiation to the melanopic efficacy of luminous radiation of D65. Melanopic DER is unitless and ranges from ~0.40 to 1.60. EML and mel-EDI are related by a scalar multiplier, where

(3) $$\begin{array}{r} \text{EML}\approx \left(\text{mel-EDI}\right)\left(1.103\right) \end{array}$$

A limitation of the Lucas et al. and CIE methods is that they are based solely on photopigment signals, yet it is unknown how photopigment signals are combined by photoreceptors and processed by the brain. Understanding of how EML and mel-EDI relate to non-visual outcomes in real-world settings is incomplete. The CIE method is the only consensus standard for characterizing the instantaneous biological potency of a light stimulus, but it comes with the caveat that quantities derived using the CIE system may not necessarily represent how light influences non-visual responses in real-world settings.

Circadian Stimulus (CS) is a non-linear model of human nocturnal melatonin suppression that is based on the quantity and spectrum of light and assumes 1-h of exposure time (33–36). CS models a hypothesized relationship between the retina and pineal gland as operating on a spectral opponency between the “blue” and “yellow” channels, from which Circadian Light, *CL*_a_, values are determined. CS is calculated by fitting *CL*_a_ values to a four-parameter logistic function. CS is expressed as a decimal percentage of melatonin suppression. CS ranges from 0.00 (0%) to 0.70 (70%). Though CS is a measure of melatonin suppression, it is intended to be relevant for the regulation of circadian rhythms. A limitation of CS is that melatonin suppression and circadian phase shift are not proxies for each other (37), and CS does not necessarily represent how light influences other non-visual responses.

There are publicly available calculators for computing the above quantities. For a Microsoft Excel calculator to compute EML see IWBI (38). For a Microsoft Excel calculator to compute mel-EDI see CIE (39). CS can be computed using an online calculator (40) or Microsoft Excel (41). Computation of EML, mel-EDI and CS require two inputs, a light source's spectral power distribution (SPD) and photopic illuminance at the plane of the eye. In principal, these quantities can be measured on site—though the complexity and cost of doing so in a non-cursory way must be acknowledged. It should also be understood that instantaneous measurements may not be representative of mean light exposure, which is almost always transitory and varies with things such as view direction, instantaneous daylight exposure, and lamp aging factors such as lumen depreciation and spectral changes with time. There are recommendations for measurement and reporting of light exposure in experiments (42, 43), with some recommendations being transferable to field settings. Spectral lighting software is available to investigate some of these measures via simulation (44, 45).

### Responses to Light That Matter

Humans have a wide range of visual and non-visual responses to light. Human outcomes most commonly relevant in applied lighting are visual performance, visual experience, visual comfort, circadian phase-shifting, and alertness. This prioritization is similar to the response headings in Figure 1, but not identical since here we are endeavoring to be more explicit. For example, while alertness, melatonin suppression, and pupil size can all be acute responses, we believe that alertness is more tangible in daily life.

*Visual performance* refers to the ability of the eye/brain system to gather and process visual information to perform a task. *Visual experience* refers to the perceptual response to illuminated environments, including evoked perceptions and emotions such as feelings of relaxation, tension, spaciousness, closure, and the like. *Visual comfort* is a reference to the perceptual response to the qualities and quantities of light in an environment, and whether they result in comfortable or uncomfortable seeing. Since visual discomfort is usually simpler to define than visual comfort, a visually comfortable environment is often defined as one that does not create visual discomfort. *Circadian phase-shifting* refers to the ability of light to advance or delay the circadian clock, and so sleep timing. *Alertness* refers to light's potential to moderate a person's state of sensory awareness and active attention. These five categories are not exhaustive and may not receive equal prioritization in a design solution. For example, the primary goal in retail lighting may be to encourage sales (46) and in open surgery visual performance will dominate the design criteria (47).

Visual performance, visual experience, and visual comfort have undergone decades of research and are the basis for lighting industry standards, recommended practices, and design guidelines. Of the various non-visual responses to light, alertness (48, 49), melatonin suppression (50, 51), and circadian phase shifting (51, 52) have been most extensively studied, mostly in laboratory settings. Pupillary response (53, 54), heart rate (55, 56), mood state (57, 58), and body temperature (55, 56) have also been studied, as has student performance in school settings (59–62), workspaces (63–66), and senior living centers (67). In this section we summarize considerations related to circadian entrainment, alertness, and performance of students and office workers since these considerations are especially relevant to how lighting design influences human health.

#### Circadian Entrainment

*Circadian rhythms* are biological rhythms with predictable changes in magnitude that repeat on a period of about 24 h. Examples include core body temperature, alertness, and the concentration of melatonin. *Circadian entrainment* is a stable relationship between a biological rhythm and an external environmental cue. A *circadian phase shift* is a change in the timing of circadian rhythms, where a *phase-advance* means that bedtime and wake-up time will move earlier in the day, and a *phase-delay* means that bedtime and wake-up time will move later in the day. The light/ dark cycle is the most important exogenous cue for entraining circadian rhythms. Reduced contrast between day and night can weaken circadian entrainment (6).

Acute suppression of the hormone melatonin is much easier to measure than changes in circadian rhythms and has been treated as a proxy measure in some of the studies endeavoring to study the effects of light on circadian health (33, 34, 36, 68, 69). Unfortunately, acute melatonin suppression by light may not be a suitable proxy for other physiological responses, such as circadian phase shifting and alertness (37, 70). The human circadian system adapts to prior light exposure (71), light exposure earlier in the day affects the biological potency of light later in the day (72–75), extended periods under dim light may negatively impact subsequent sleep (76), and there is considerable interindividual variability in the response to evening light (77). Collectively, these findings suggest nuance in the human circadian response to light, raising questions about the veracity of numerical design targets for circadian lighting design. For example, the CS targets in UL 24480 (10) are based on research that characterizes acute melatonin suppression after a 1-h exposure to light, yet compliance with the UL standard requires a minimum of a 2-h exposure of CS > 0.30 between 7 a.m. and 4 p.m., and is intended to support a broad range of health outcomes for all day-shift workers.

Daylight naturally provides bright days and dark nights, creating both the cycle and light/dark ratio that is essential for circadian entrainment (78, 79). Daylight offers other psychological and physiological benefits (80–82) and, whenever possible, should play a key role in human-centric lighting design (83).

#### Alertness

The potential to use light to enhance alertness and improve cognitive performance is of interest to educational institutions and knowledge-work environments. Organizations that operate 24/7 may also aspire to reduce errors and increase safety by using light to enhance nighttime alertness of employees.

The alerting response has largely been characterized in laboratory settings during nighttime. The acute alerting properties of light have been compared to that observed after caffeine consumption (84). In laboratory settings, light has reduced attentional lapses, decreased subjective sleepiness, improved alertness, and enhanced performance on some cognitive tests (11, 15, 85–87). The alerting effects of light may be stronger at night than during the day (87). During daytime hours, improvements in alertness may be minimal for well-rested people (88). Many studies of alertness and cognition have shown mixed results where some measures have improved but others have not (15, 89–92).

Alertness is commonly characterized using an objective assessment of sustained attention (93) or a subjective alertness measure (94). An advantage of these standardized tests is that they are sensitive to sleep deprivation and have a large base of prior literature for comparison. More complex tasks, however, may be resistant to the short-term alerting effects of light. The generalizability of simplistic assessments of attention to real-world outcomes is questionable.

In their review, Souman et al. (15) concluded that increasing the intensity of white light sometimes increases subjective ratings of alertness, that the effect of CCT on subjective alertness is unclear, and that no studies show a systematic relationship between alertness and wavelength. The meta-analysis by Brown (95) suggests a sigmoidal relationship between subjective sleepiness and mel-EDI (18). Others have shown that light without short-wavelength content can maintain subjective alertness without suppressing melatonin (96, 97), and that long-wavelength (red) light can elicit an alerting response during daytime hours without acute melatonin suppression (98). Few studies have tested the potential alerting benefits of architectural lighting relative to operational outcomes in real-world settings. Despite the consensus that light influences alertness, especially in controlled settings, and general guidance in the peer-reviewed literature (99), no consensus body has offered lighting criteria that implies a direct stimulus response relationship between light and alertness.

#### Student and Office Worker Performance

There is some evidence that lighting influences student performance. Early evidence demonstrated a positive connection between the presence of daylight in classrooms and student performance (100). More recently, classroom lighting with relatively more short-wavelength radiation was shown to improve cognitive processing speed in high school students (59), improve concentration in elementary school children (60), and improve oral reading fluency performance in third-grade students (61). Lighting that varies in color temperature and illuminance was shown to increase attention and reading speed of elementary and high school students (62). Collectively, these studies suggest that architectural lighting, including daylight, has the potential to positively impact student learning.

There is also some evidence that lighting influences knowledge worker performance. Lighting with a very high CCT of 17,000 K was employed within a shift-working call center (63) and in an office setting (64). Mills et al. found mean improvements on fatigue, alertness, daytime sleepiness, and work performance. Viola et al. found mean improvements on alertness, mood, daytime sleepiness, evening fatigue, and work performance. Office workers in windowless environments self-reported poorer well-being and sleep quality in comparison to workers with access to windows (65). View very likely plays a role, but the reported positive effects of daylight and view have not yet been disentangled (101). Figueiro et al. (66) linked daytime light exposure to the sleep quality and mood of office workers. Collectively, these studies suggest that architectural lighting, including daylight (and view), has the potential to influence office worker well-being and performance.

### External Validity

External validity is the extent to which results can be applied to other people or contexts that differ from the specific circumstances of the experiment (102). Outside of laboratory settings, responses to light do not occur in isolation. The same light stimulus may simultaneously influence none or many biological responses. A biological response may or may not translate to a change in performance or overall health. Any of the outcomes mentioned above can be influenced by factors other than light, such as age, climate, diet, disease, exercise, genetics, medications, mental health, pregnancy, sleep habits, stress, and travel. Responses evoked by light may also be evoked by other sensory inputs, such as an attention response evoked by auditory (103) or olfactory (104) stimuli. While light is indeed potent, it is important to consider the effects of light in a broader context that includes other sensory and non-sensory inputs.

Of the field studies available, many have used participants with limited or less-common exposure to light, such as elders with limited mobility (67, 105–107), night shift workers (108–111), fatigued cancer survivors (112), hospitalized patients (113), and infants (114). In most of these studies, the benefit of lighting was modest or the outcome was mixed.

The least studied group are healthy adults that work during the day, sleep at night, and who have exposure to daylight. For this group, if electric light exposure is compliant with guidelines for vison (26, 115), it is unclear the degree to which changing the lighting will affect non-visual outcomes. While laboratory studies have demonstrated the capacity and potential of light to influence non-visual outcomes, more field studies are needed to understand the veracity of the effects in real-world settings.

## A Five-Step Design Process for Human-Centric Lighting

Though there is still much to learn, enough is known today to at least offer a framework for addressing visual and non-visual outcomes through lighting design decisions. The five-step process outlined below augments the already well-established lighting design processes (116) and can be used to integrate human-centric lighting design concepts into design practice. Table 2 provides an overview of the process with examples for select application types.

**Table 2:** A sample of representative application-specific examples.

**Application**	**Characteristics**	**Step 1**	**Step 2**	**Step 3**	**Step 4**
		**Operational goals**	**Likely occupant sleep-wake cycle**	**Occupant sleep needs**	**Human-centric lighting principles^a^^,^^b^**
Military and Maritime	• Demanding environment with low tolerance for errors	• Safety • Achieve mission objectives • Maximize energy efficiency • Maximum system reliability	• Applications are likely to have both day-active and night-active people, or both simultaneously	• Application is likely to have a mix of sleeping and active occupants	A, B, C, D, E, F
Healthcare	• Environments intended to prevent, cure, or treat illness	• Safety • Save lives • Improve patients' quality of life • Minimize suffering	• Patients: likely to be day-active, but may be night-night active as well • Care providers: doctors and nurses are likely to be both day-active and night-active depending on the shift and the type of healthcare environment	• Application is likely to have a mix of sleeping and active occupants • Sleeping occupants consists of patients using inpatient services	A, B, C, D, E, F
Hotel	• Strong need for aesthetic considerations and brand-conscious design	• Create mood and atmosphere consistent with brand identity • Accommodate guest sleeping and waking needs	• Guests: Quite variable, with many suffering from jet lag • Employees: 24/7 operation requires some day and some night workers	• Guest have a variety of sleep needs due to circadian phase shifts from different time zones	B, D, E
Education	• Environments dedicated principally to teaching and learning	• Learning	• Mostly day-active people, though likely working/studying into evening hours	• Application is unlikely to have sleeping occupants	A, B, C, D, E
Industrial and Commercial	• Productivity is important • May be non-specific productivity, such as increasing attentiveness of office workers • May be task-specific productivity, such as minimizing assembly line errors	• Safety • Productivity	• Day-active • Some applications, such as 24/7 industrial facilities, may include night-active workers	• Application is unlikely to have sleeping occupants	A, B, C, D, E, F

*There could be many sub-categories within each row that are not shown*.

a *All applicable codes and standards must also be addressed, including those related to safety and energy*.

b *Refer to Table 3 for published WELL v2 and UL guidelines*.

**Key for human-centric lighting principles**

***A** Comply with recommended practice for light level and quality, as from CIBSE (115) or IES (26)*.

***B** Address lighting quality (e.g., low glare, no flicker, good color rendition)*.

***C** Maximize daylight exposure/outside view while controlling for possible glare from the sun and sky*.

***D** Consider psychological reinforcement (e.g., positive distraction in healthcare, color tuning in classrooms, aesthetics in hotels)*.

***E** Evaluate/consider WELL and/or UL guidelines for day-active people where applicable*.

***F** Provide light to promote visual performance for nighttime activities where applicable*.

### Step 1: Characterize the Lighting Application

The first step is to establish the application's primary tasks and activities, when they occur, and the desired outcomes or operational goals. For example, healthcare environments are typified by the intent to prevent, cure, or treat illness. Operational goals will likely include improving patient health and well-being, which often requires visual evaluation, diagnostic testing, and interpersonal communication. Architectural design and human-centric lighting strategies should support these activities.

A thorough understanding of desired outcomes will guide prioritization of design criteria and facilitate rational design decisions when all outcomes cannot be simultaneously achieved. For example, in a healthcare environment, temporary visual discomfort of a patient may be acceptable if it increases the speed and efficacy with which a caregiver can diagnose and administer treatment (e.g., over-bed patient exam lights tends to be very bright when viewed from the perspective of a patient, but are necessary for the caregiver). In an office environment, such visual discomfort would likely affect productivity and would therefore be unacceptable. There is no one-size-fits all approach.

### Step 2: Determine the Likely Sleep-Wake Cycle(s) of Occupants

Determine if the application includes day-active people, night-active people, or both. Occupants that are day-active/night-inactive have wake-sleep cycles that are largely synchronous with the day-night cycle. Their lighting needs may conflict with occupants that are night-active/day-inactive and who have wake-sleep cycles that are largely asynchronous with the day-night cycle. Day-active people benefit from light with high biological potency during the morning and daytime, low biological potency in the evening, and as little biological potency as possible at night. Night-active people need nighttime illumination to adequately and safely perform personal or professional tasks while minimizing the potential negative human outcomes associated with an asynchronous sleep-wake cycle and nighttime light exposure.

For example, education settings are likely to have predominately day-active occupants, 24/7 industrial facilities may have day-active or night-active people sequentially throughout the day as the work shift changes from day-shift, to second-shift, to night-shift, and healthcare facilities may include both day-active and night-active occupants simultaneously. Applications with both day-active and night-active people—either sequentially or simultaneously—may require specialized design solutions that include advanced lighting controls (e.g., controlling zones, scenes, intensity, spectrum) and/or architectural interventions (e.g., barriers to block obtrusive light, temporary or permanent partitions to create zonal workspaces) that rethink traditional architectural and spatial relationships.

It is especially challenging to address the needs of workers with rotating shifts, such as nurses that cycle between dayshifts and nightshifts. During periods of rotation, the circadian pacemaker is playing perpetual catch-up to the changing timing of the light exposure rhythms. The optimal lighting solution for an occupant with a rotating shift may differ from the optimal lighting solution for an occupant with a permanent shift, even when both people occupy the same environment at the same time and are performing comparable work tasks.

### Step 3: Determine the Sleep Needs of Occupants

Determine if the application includes sleeping occupants. If yes, determine if the sleeping occupants will be day-active, night-active, or both. Darkness promotes sleep and spaces where occupants will sleep should be as dark as possible while providing enough light for safe navigation (27). For day-active people sleep occurs primarily in the evening and throughout the night—this may require window treatments to block intrusive light. For night active people, sleep occurs primarily during daytime hours and window treatments or eye coverings are almost certainly necessary.

For example, healthcare environments are likely to have a combination of occupant needs: day-active (or night-active) patients seeking treatment during the day (outpatient); day-active (or night-active) patients seeking treatment over several days (inpatient), with both active and sleep requirements; and the healthcare workers that provide care, and who may be day-active or night-active, depending on the shift. These groups have separate and distinct needs that change throughout the day and the interplay between conflicting needs by different occupants demands careful examination, including prioritization and a considered understanding of tradeoffs. See Zee and Goldstein (117) for guidance about using light and sleep hygiene practices to improve outcomes for people with non-traditional work schedules.

### Step 4: Review Published Human-Centric Lighting Guidance

Human-centric lighting design can be informed by industry guidelines that endeavor to bridge the gap between scientific understanding and guidance for application. Organizations like the Society for Light and Lighting and the Illuminating Engineering Society (IES) provide standards that focus of visual outcomes (26, 115). The WELL Building Standard (WELL) (9) and Underwriters Laboratory (UL) (10) each recommend quantitative design targets for circadian lighting design. The WELL standard has changed threshold values over time; to our knowledge, the rationale for the threshold design targets and changes to the thresholds has never been disclosed. The IES maintains that, to ensure transparency and involvement of relevant constituencies, any recommended practice related to light and health should be a consensus document developed through an accredited ANSI process (118), which was not done with either the WELL or UL standards. DIN SPEC 67600 also provides design guidelines for biologically effective illumination (119). DIN SPEC 67600 was not developed through the full ISO consensus standards process, which is why it bears the “SPEC” modifier in the title (120).

The WELL criteria are based primarily on EML (32), while also allowing compliance with mel-EDI (18) or CS (36). UL's criteria are primarily expressed using CS while also allowing compliance using EML or photopic illuminance. Both WELL and UL have exposure times and durations associated with their recommendations. The UL standard is limited to promoting circadian entrainment for day-active and night-inactive people in commercial, educational, and industrial settings, while encouraging consideration of other legitimate design goals such as glare and color quality. The WELL standard is also intended to promote circadian entrainment, while also including explicit pass/fail criteria related to illuminance, glare, visual comfort, access to daylight, views, color quality, flicker, and personal control. Both systems endeavor to promote a comprehensive approach to human-centric lighting. The criteria related to circadian entrainment for the UL and WELL systems are provided in Table 3. The criteria are based on the temporal pattern of light exposure (time of day and duration of exposure), light level, spectrum, and the location where the light is delivered. While Table 3 is current as of the date of this article, it is prudent to check for updates since recommendations may change.

**Table 3:** Guidelines published by the WELL Building Standard v2 and Underwriter's Laboratory 24480.

**Standard**	**Temporal pattern**		**Lighting quantity (Note: these are a function of light level and spectrum)**				**Location**
	**Timing of exposure**	**Duration of exposure**	**Circadian stimulus (CS) (Percent)**	**Equivalent melanopic Lux (EML) (Melanopic Lux)**	**Melanopic equivalent daylight illuminance (Melanopic EDI) (Lux)**	**PhotopicIlluminance (Lux)**	
WELL v2.0Requirements for 1 point	At least between the hours of 9 a.m. and 1 p.m. Light levels may be lowered after 8 p.m.	Minimum of 4 h.	≥0.30 (if electric light only)	≥150 (if electric light only)≥120 from electric lighting (if certain daylighting criteria are met)	≥ 136 (if electric light only)≥109 from electric lighting (if certain daylighting criteria are met)	N/A	Vertical plane at eye level
WELL v2.0Requirements for 3 points	At least between the hours of 9 a.m. and 1 p.m. Light levels may be lowered after 8 p.m.	Minimum of 4 h.	N/A	≥240 (if electric light only)≥180 from electric lighting (if certain daylighting criteria are met)	≥ 218 (if electric light only)≥163 from electric lighting (if certain daylighting criteria are met)	N/A	Vertical plane at eye level
UL 24480	7 a.m.−4 p.m.	Minimum of 2 h, morningIf not full period	≥0.30	Comply with WELL criteria shown above based on desire for 1 point or 3 points	N/A	≥500	Vertical plane at eye level
	5–7 p.m.	During full period	≤ 0.20		N/A	N/A	
	After 8 p.m.	During full period	≤ 0.10		N/A	N/A	

*Note that where alternative compliance paths are offered, either CS, EML, EDI, or Photopic Illuminance can meet the criteria*.

Brown et al. (27) provide recent and noteworthy light exposure recommendations for healthy adults with regular daytime schedule. They recommend mel-EDI ≥ 250 lx throughout the day, mel-EDI ≤ 10 lx in the 3 h before bedtime, and sleep environments as dark as possible (mel-EDI ≤ 1 lx). All measurements are at the plane of an observer's eye, simplified as a vertical plane at ≈ 1.2 m height. Insofar as possible, these recommendations should be applied daily at the same time of day. These recommendations are not intended to supersede existing guidelines related to visual function and safety; rather, they are additional criteria to be considered by lighting specifiers. The daytime criteria of Brown et al. (27) are higher in quantity and longer in duration than both the WELL and UL criteria.

WELL v2 (9), UL 24480 (10), and Brown et al. (27) provide pass/fail criteria that facilitate ease-of-use in practice but may promote a false sense of precision. Considerations of precision and accuracy are relevant (121) since they can complicate the debate about how to establish metrics and thresholds that serve both vision and autonomic body functions that are influenced by light. It is already appreciated that under many circumstances great precision is not needed when designing lighting systems. The IES Lighting Handbook (26) suggest that, at time of occupancy, illuminance measurements within 30% of the target are sufficiently accurate for most applications. Similar considerations are likely relevant for non-visual effects, since physiological responses are modulated by large relative changes more so than by small fractional changes. The key considerations in Well v2 (9), UL 24480 (10), and Brown et al. (27) focus on defining the magnitude of photic stimulation at the plane of the eye, linked to time of day, for a given length of time. While the recommendations available today are not identical, they are comparable in manner and degree, which we view as progress toward consensus. The fuzziness that exists today is not a problem since estimates need not be overly precise or accurate to be useful in lighting practice. We believe WELL v2 (9), UL 24480 (10), and Brown et al. (27) all move lighting practice in a positive direction by encouraging consideration of non-visual responses to light and by providing quantitative target that can inform the development of design criteria.

### Step 5: Put It All Together

Once the application characteristics and operational goals have been defined, the occupants' sleep-wake cycles established, occupant sleep requirements have been determined, and published guidance has been reviewed, design criteria and numerical design targets can be established. Recommendations from WELL, UL, CIBSE, and IES provide guidance, but it is ultimately the duty of the design team, in consultation with the building owner, to balance the relative importance of visual and non-visual needs. The WELL and UL circadian lighting guidelines are specifically for day-active (night-inactive) people; stakeholders will need to use their best judgement when designing lighting systems for people who do not fit this profile or where multiple populations or people with different schedules occupy the same spaces.

Lighting design demands consideration of competing criteria. Prioritization may be needed to balance tradeoffs between visual and non-visual design goals. For example, bright light during the day is expected to better support non-visual outcomes for day-active people. But, if the light is so bright that it creates glare, then productivity may suffer. Higher light levels also require more energy, which may not be compatible with required energy codes (122). As another example, darkness is most desirable at nighttime for circadian health, but light may be needed to support safe navigation. Such difficult tradeoffs are best addressed by competent professionals and thoughtful clients that are willing to prioritize needs and define expected outcomes.

Design teams work on projects on a per-project basis, where a typical project may be a building or a subset of spaces within a building. Yet, people transition through many buildings and spaces through the course of a day, week, month, and year. Coworkers or classmates may have very different spectral diets (123), which will moderate the manner with which comparable workplace or school lighting affects individual outcomes. Individuals have different levels of control over their exposure to light. Some people may be able to take morning and lunchtime walks and limit screen use at night. Others may have limited mobility or inflexible work schedules. Even longitudinal position within a time zone affects cancer risk, likely due to varying degrees of circadian disruption (124). Though the design team cannot control these and other pertinent factors, designers have some control over the light stimulus received by occupants in the spaces they design. By providing light with appropriate qualities (e.g., intensity, spectrum, spatial pattern, controllability) at the right time of day, designers can support good outcomes even against this background of uncertainty.

## Conclusions

For day-active people, visual and non-visual needs are generally synchronized. While light from the sun and sky naturally provides the cycle and light/dark ratio that supports circadian health, electric lighting has a critical role since most of us spend most of our time indoors (4, 5). Architecture, glazing, lighting equipment, and lighting control solutions can be used in combination to deliver biologically potent light during the day while minimizing light exposure as night, all while balancing traditional factors such as color quality, flicker, glare, psychological reinforcement, and visibility (26, 115). Perhaps the simplest guidance to support the health of day-active people is to provide light of high biologically potency during the day and low biological potency at night.

For night-active people, visual and non-visual needs are in conflict, requiring explicit prioritization. Many night-active people provide critical societal functions, such as night shift nurses and doctors, with visually demanding jobs that have a low tolerance for errors. For these people, factors like visibility and alertness may be prioritized over circadian entrainment, even while recognizing that circadian disruption from an asynchronous sleep-wake cycle is associated with long-term negative health outcomes (125). Lighting design solutions for night-active people demands complete consideration of tradeoffs.

Good outcomes are most likely when a knowledgeable team that includes designers, owners, and equipment manufacturers prioritize visual and non-visual design outcomes. Such teams are well-positioned to develop lighting solutions that deliver light of an appropriate amount and spectrum at the right time of day, for an appropriate length of time.

Because there is no one-size-fits-all solution, we suggested a framework to guide lighting design: characterize the lighting application, determine the likely sleep-wake cycle(s) of occupants, determine the sleep needs of the occupants, review published guidance to develop goals and design criteria that support visual and non-visual outcomes, then use this information to establish design criteria that will guide decisions in the latter stages of the design process. We hope that implementation of this process facilitates realization of what we all want—lighting solutions that support human outcomes.

## Author Contributions

KH and TE contributed to conceptualization, writing, reviewing, and editing of this manuscript, including figures and tables. KH managed project administration. All authors contributed to the article and approved the submitted version.

## Conflict of Interest

KH is Editor in Chief of LEUKOS, the journal of the Illuminating Engineering Society, founder of Loucetios, LLC, and co-founder of Lyralux, Inc. TE is founder of Lighting Research Solutions, LLC.
